# Validation of the Caprini risk assessment model and efficacy of perioperative nutritional support + preventive nursing in reducing DVT among high-risk older adults undergoing hip fracture surgery: a two-phase study

**DOI:** 10.3389/fmed.2026.1680122

**Published:** 2026-03-02

**Authors:** Pingfang Liu, Ting Shu, Manhua Liu, Fei Liu, Yongping Li

**Affiliations:** 1 Department of Nursing, Hunan University of Medicine General Hospital, Huaihua, Hunan, China; 2 Graduate School, Jishou University School of Medicine, Jishou, Hunan, China; 3 Department of Orthopedics, Hunan University of Medicine General Hospital, Huaihua, Hunan, China

**Keywords:** Caprini risk assessment scale, deep venous thrombosis, elderly, hip fracture, nutritional support, preventive nursing

## Abstract

**Objective:**

To evaluate the predictive accuracy of the original Caprini risk assessment model for postoperative deep venous thrombosis (DVT) in older adult patients with hip fractures and to assess the effect of perioperative nutritional support combined with preventive nursing on nutritional status and DVT incidence.

**Methods:**

A total of 112 older adult patients with hip fractures who underwent surgical treatment were included. Patients were retrospectively categorized as DVT (*n* = 38) or non-DVT (*n* = 74) groups for Phase 1 analysis. For Phase 2, all patients were prospectively randomized into a control or intervention group (*n* = 56 each). The predictive accuracy of the original Caprini scale was analyzed using receiver operating characteristic (ROC) curves. The intervention group received preventive nursing (based on original Caprini scores) and perioperative nutritional support, while the control group received routine care. Nutritional status, functional outcomes, DVT incidence, and satisfaction were compared.

**Results:**

The original Caprini score was higher in the DVT group (*p* < 0.05). Using the original classification (low: 0–1, moderate: 2, high: 3–4, highest: ≥5), the proportion of patients in the “highest risk” category (≥5) was significantly higher in the DVT group (94.74%) than in the non-DVT group (70.27%) (*p* < 0.05). The AUC was 0.724 (95% CI: 0.657–0.791; *p* < 0.001). Postoperatively, the intervention group showed superior nutritional biomarkers (ALB, PA, Hb), shorter time to mobilization and hospital stay, better hip function (Harris score) and quality of life (SF-36), a lower DVT incidence (*p* < 0.05), and higher nursing satisfaction (*p* < 0.05).

**Conclusion:**

The Caprini risk assessment model demonstrates predictive value for DVT risk in this population. The combined intervention of perioperative nutritional support and preventive nursing improves nutritional status, functional recovery, and reduces DVT incidence.

## 1 Introduction

Hip fractures refer to fractures occurring from the femoral head to the lesser trochanter (>5 cm), including femoral neck, intertrochanteric, and subtrochanteric fractures (1). Hip fractures usually occur after a fall and are among the most common osteoporotic fractures in the older adults, with a substantial impact on patients’ quality of life (2). With the acceleration of social development and population aging in China, the incidence of hip fractures in the older adults is expected to increase proportionately (3). Deep venous thrombosis (DVT) is the most common and serious complication of hip fracture (4). Previous studies have reported that the incidence of perioperative DVT due to hip fractures ranges from 11.1 to 34.98% (5). Besides, it has been reported that the prevalence of postoperative DVT following hip fractures was 32.8% (6). DVT not only delays recovery and prolongs hospitalization but may also lead to pulmonary embolism if the thrombus dislodges, which can be life-threatening (7). Therefore, early prevention and accurate risk identification are essential to reduce DVT incidence.

The Caprini risk assessment model is an effective clinical tool for quantitatively predicting the risk of venous thromboembolism (8). Relevant guidelines recommend its use for DVT risk assessment in patients undergoing major orthopedic surgery (9). Numerous studies have further validated its clinical application value. For instance, Zhang et al. demonstrated in their multi-center retrospective cohort study that the prevalence of DVT and higher Caprini score were significantly associated with increased all-cause mortality among orthopedic trauma patients after discharge (10). Sun et al. indicated that the risk group classification by Caprini RAM was significantly associated with preoperative DVT (11).

In addition, the correct application of the nursing model is particularly important for patients’ postoperative recovery (12). The preventive nursing model is a new approach that can evaluate nurse-related risks in advance and formulate corresponding measures based on the evaluation results, playing a crucial role in preventing DVT (13).

Malnutrition is not only an important cause of hip fractures in older adult patients but also a major factor affecting prognosis (14). Nutrition survey data show that at least half of hospitalized older adult patients are malnourished (15). After a hip fracture, elevated catabolism and reduced dietary intake further worsen nutritional status, resulting in weight loss, muscle atrophy, bone mass loss, and other complications that adversely affect postoperative fracture rehabilitation (16). Adequate nutritional support has been shown to be crucial for improving patient outcomes. Studies by Sarkies et al. indicated that early and appropriate nutritional interventions could significantly reduce the incidence of complications, shorten the length of hospital stay, and enhance the overall recovery of patients with hip fractures (17). Moreover, Jiménez et al. found that an oral nutritional supplement could effectively improve the nutritional status of older adult patients with hip fractures (18). Therefore, improving the nutritional status of older adult patients with perioperative hip fractures is closely associated with their prognosis.

Therefore, improving the nutritional status of older adult patients with perioperative hip fractures is closely associated with their prognosis.

In this study, we aimed to explore the application of postoperative thrombosis risk assessment using the Caprini risk assessment model in older adult patients with hip fractures and the effect of perioperative nutritional support combined with preventive nursing on nutritional status and DVT in older adult patients undergoing hip fracture surgery.

## 2 Data and methods

### 2.1 Study design and protocol

This was a prospectively designed, two-phase, integrated cohort study conducted under a single, predefined research protocol. The protocol explicitly outlined two primary, sequential objectives with distinct analytical approaches to avoid conceptual and statistical conflation:

#### 2.1.1 Phase 1 (observational/diagnostic)

To validate the predictive accuracy of the Caprini Risk Assessment Model for postoperative DVT in older adult patients undergoing hip fracture surgery.

#### 2.1.2 Phase 2 (interventional/randomized controlled)

To evaluate the efficacy of a combined perioperative nutritional support and risk-stratified preventive nursing intervention on improving nutritional status, functional recovery, and reducing DVT incidence within the same cohort.

While the occurrence of postoperative DVT is assessed as an endpoint in both phases, the analytical inferences are independent. Phase 1 evaluates the diagnostic accuracy of the Caprini score against this endpoint. Phase 2 evaluates the efficacy of the intervention by comparing DVT incidence between randomized groups. The randomization for Phase 2 ensures that any association found in Phase 1 between Caprini score and DVT does not bias the estimation of the intervention effect in Phase 2.

#### 2.1.3 Sample size and power calculation

This study was designed as a confirmatory randomized controlled trial (RCT) for its primary Phase 2 objective. The sample size calculation was prospectively performed for the Phase 2 RCT using G*Power software (version 3.1). The calculation was based on detecting a clinically meaningful difference in the primary nutritional outcome—serum albumin level at postoperative day 7—between the control and intervention groups. Based on previous literature and clinical expertise, we assumed a standardized effect size (Cohen’s d) of 0.5. Using a two-tailed independent *t*-test with an alpha (*α*) level of 0.05 and a beta (*β*) level of 0.20 (statistical power of 80%), the calculation indicated a requirement of approximately 51 patients per group. To account for potential attrition and to ensure sufficient power for the Phase 1 diagnostic accuracy analysis, we planned to recruit a total of 112 patients (56 per group). This larger sample also ensured an adequate number of positive events (DVT cases) for a reliable Phase 1 ROC curve analysis, which typically requires at least 50–60 events for stable AUC estimation. Our final sample of 112 patients with 38 DVT events fulfilled the recruitment target for the RCT and provided a sufficient number of events for the diagnostic validation component.

#### 2.1.4 Statistical analysis plan to address multiplicity and dependence

The analysis plans for the two phases were pre-specified as distinct.

For Phase 1, diagnostic accuracy was assessed using ROC analysis. Comparisons between the DVT and non-DVT groups (derived from postoperative outcome) on Caprini scores and other baseline variables used chi-square or *t*-tests as appropriate.

For Phase 2, all efficacy analyses adhered to the Intention-to-Treat (ITT) principle, based solely on the randomization assignment from this phase. Between-group comparisons for continuous outcomes used independent *t*-tests or Mann–Whitney U tests, and for categorical outcomes (including DVT incidence), used chi-square tests. The analysis of DVT incidence in Phase 2 was treated as a secondary, exploratory comparison without adjustment for multiple testing across secondary outcomes, as predefined in the protocol. This clear separation of analytical frameworks mitigates the risk of overinterpretation or bias from using shared data.

### 2.2 Study population, grouping, and bias control

A total of 112 older adult patients with hip fractures who underwent surgical treatment at our hospital between January 2022 and December 2023 were included in the study.

#### 2.2.1 Analytical grouping for phase 1 (model validation)

To assess the predictive accuracy of the Caprini risk assessment model (Phase 1), the entire cohort was retrospectively categorized post-hoc based on the postoperative clinical outcome. Patients were divided into two groups: the DVT group (*n* = 38) and the non-DVT group (*n* = 74). The Caprini scores, collected at admission, were then compared between these groups to evaluate the model’s discriminatory ability.

#### 2.2.2 Randomization and grouping for phase 2 (intervention trial)

**Figure 1:**
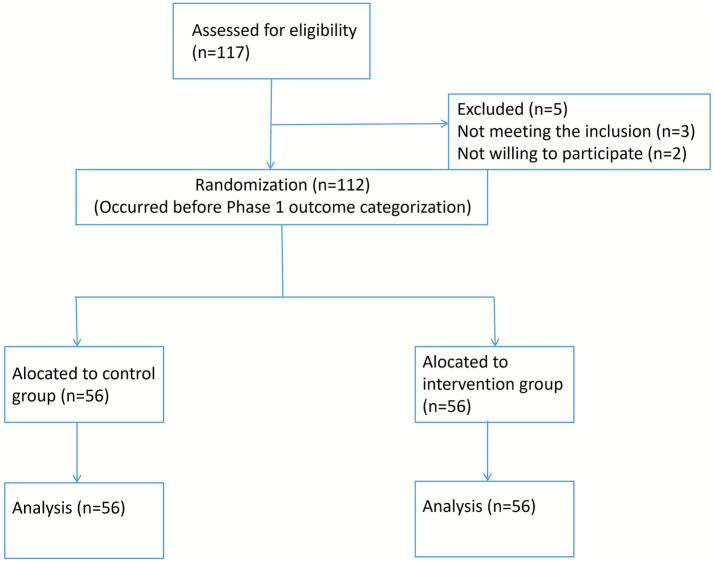
CONSORT-style flow diagram.

For the evaluation of the intervention’s efficacy (Phase 2), all 112 enrolled patients were prospectively randomized. Using a computer-generated random number sequence, patients were allocated 1:1 to either the control group (*n* = 56) or the intervention group (*n* = 56). This allocation was performed independently of and prior to the determination of their final DVT status in Phase 1, ensuring that the randomized comparison for intervention effects was unbiased by the outcome. The flow of participants is detailed in Figure 1.

#### 2.2.3 Allocation concealment

Allocation concealment was strictly maintained using sequentially numbered, opaque, sealed envelopes (SNOSE). An independent research assistant, not involved in participant recruitment, clinical care, or outcome assessment, prepared the envelopes and managed the allocation sequence. Upon a patient’s confirmed eligibility and provision of informed consent, the treating clinician contacted this assistant, who then opened the next sequential envelope to reveal the group assignment. This process ensured that investigators involved in enrollment could not foresee the allocation, minimizing selection bias.

#### 2.2.4 Blinding procedures and management of potential biases

We acknowledge that due to the nature of the complex behavioral and educational intervention, complete blinding of patients and the nursing staff (caregivers) delivering the care was not feasible. This introduces a risk of performance bias. To mitigate this risk, we implemented the following strategies:

1. *Standardization of core care*: All patients, regardless of group assignment, received identical standard surgical treatment, routine postoperative monitoring (vital signs, limb checks), and basic mobilization protocols. This ensured a common baseline of care.
2. *Caregiver training and protocol adherence*: Nurses delivering the intervention underwent rigorous, standardized training on the study protocol. They were instructed to interact with all patients in a professional and supportive manner, focusing on the execution of prescribed tasks rather than differential attitudes. Adherence to the specific intervention components (e.g., duration of pump use, exercise guidance) was monitored using checklists.
3. *Blinding of outcome assessors*: Crucially, all outcome assessors were blinded to group allocation. This team was entirely separate from the clinical care and randomization team. Assessments for nutritional biomarkers (Hb, PA, ALB), DVT diagnosis via Doppler ultrasound, Harris Hip Score (performed by an independent physiotherapist), and SF-36 quality of life questionnaire scoring were conducted by personnel unaware of whether the patient was in the control or intervention group. The ultrasound technicians were provided with clinical indications only, not group assignment.
4. *Objective outcome prioritization and adjudication*: To further minimize detection bias, we pre-specified a hierarchy of outcomes, prioritizing objective measures. The primary outcome (nutritional biomarkers) and key secondary outcomes like DVT incidence (objectively diagnosed by ultrasound) and time to independent ambulation (a clearly defined, observable milestone) were considered less susceptible to assessor bias. For the subjective patient-reported outcomes (SF-36, nursing satisfaction questionnaire), we used validated instruments and emphasized the blinding of the data analyst during the initial scoring and data entry phase.

#### 2.2.5 Analysis principle

All analyses for Phase 2 were conducted according to the intention-to-treat (ITT) principle. All 112 randomized patients were analyzed in the groups to which they were originally assigned. During the study period, there were no crossovers between groups and no patient withdrawals, ensuring a complete dataset for the ITT analysis.

### 2.3 Diagnostic criteria

The diagnosis of DVT was based on a comprehensive assessment that combined clinical symptoms and Doppler ultrasound examination. Clinical symptoms: Patients were closely monitored for typical symptoms of DVT, such as unilateral lower limb swelling, pain, tenderness, and skin discoloration (usually redness or cyanosis). Doppler ultrasound examination: DVT were identified as hypoechoic or anechoic masses within the vein lumen, often accompanied by a lack of compressibility of the vein.

### 2.4 Inclusion and exclusion criteria

Inclusion criteria: (1) Patients met the diagnostic criteria for hip fracture and were diagnosed with femoral neck fracture and femoral trochanter fracture by imaging; (2) age ≥60 years; (3) unilateral fracture and able to walk normally before injury; (4) the patient underwent hip surgery for the first time; (5) American Society of Anesthesiologists level I to III; (6) preoperative color Doppler ultrasonography showing deep veins unobstructed and without DVT; (7) normal communication and comprehension.

Exclusion criteria: (1) combined with other serious fractures; (2) coagulation dysfunction; (3) history of thromboembolism; (4) malignant tumors or severe dysfunction of vital organs.

### 2.5 Caprini risk assessment model

According to the original, validated Caprini scale scoring standard, 40 risk factors were scored based on each patient’s condition and classified into four grades: 0–1, low risk; 2, moderate risk; 3–4, high risk; and ≥5, highest risk. All analyses in this study were performed using this original classification.

### 2.6 Nursing methods

#### 2.6.1 Description of interventions

##### 2.6.1.1 Control group: routine standard care

All patients received standardized surgical treatment. Postoperative nursing followed the hospital’s routine protocol for hip fracture patients, which included:

*Preoperative*: Patient education regarding the disease and surgery; assistance with routine preoperative tests.

*Postoperative*: Monitoring of vital signs and the surgical limb (skin temperature, color, arterial pulse, swelling, pain); reporting abnormalities to physicians.

*Prophylaxis*: Pharmacological thromboprophylaxis was administered as per the standard practice of the attending surgical team, without a mandatory institutional protocol. The choice of agent, dose, and duration was at the physician’s discretion, primarily based on individual patient assessment of bleeding risk, renal function, and weight. Commonly prescribed regimens included:

*Low-Molecular-Weight Heparin (LMWH)*: Typically enoxaparin 20 mg or 40 mg subcutaneously once daily, initiated 12–24 h postoperatively.

*Fondaparinux*: 2.5 mg subcutaneously once daily, initiated 6–8 h postoperatively.

*Warfarin*: Dosed to achieve a target INR of 2.0–3.0, often initiated pre- or postoperatively.

The specific agent, dose, and time of first administration were recorded in the medical chart. The intended duration of prophylaxis was typically until hospital discharge or for a minimum of 10–14 days, but actual adherence post-discharge was not actively monitored by the study. Non-pharmacological measures included the application of graduated compression stockings (GCS) and intermittent pneumatic compression (IPC) devices as ordered. Mobilization: Limb elevation (15–30°); guidance on early ambulation (as tolerated) and postoperative precautions (e.g., avoiding crossing legs).

*Intervention group*: Caprini-based preventive nursing & perioperative nutritional support. This multicomponent intervention was delivered in addition to the Routine Standard Care described for the control group.

(A) *Core component 1*: Risk-stratified preventive nursing. This component was delivered by a trained nursing team and was tailored based on each patient’s original Caprini risk category assessed at admission.

*Staff training and competency*: All nurses completed a 4-h standardized training program. Training included: (i) theoretical knowledge of the original Caprini model and DVT pathophysiology; (ii) practical skills for scoring and risk categorization (Low: 0–1, Moderate: 2, High: 3–4, Highest: ≥5); (iii) case-based application; and (iv) certification via a written/practical test.

##### 2.6.1.2 Intervention protocol by risk category

*All patients (Baseline)*: Standard monitoring and GCS/IPC as per control group.

*Moderate risk (Score = 2)*: *Added*: (i) Daily one-on-one DVT education for patient/family; (ii) daily viewing of standardized educational videos; (iii) collaborative creation of a post-discharge rehabilitation plan.

*High risk (Score = 3–4)*: *Added to Moderate Risk measures*: (i) Twice-daily plantar venous pump therapy (10–15 min/session); (ii) limb circulation assessment every 4 h and circumference measurement every 12 h; (iii) pharmacological prophylaxis per physician order (e.g., LMWH 40 mg SC daily or warfarin with INR monitoring).

*Highest risk (Score ≥5)*: *Added to High-Risk measures*: (i) Immediate referral for lower-extremity venous ultrasound upon any clinical suspicion of DVT; (ii) activation of hospital DVT response protocol if diagnosis confirmed.

*Flexibility/Adaptation*: The specific timing for initiating GCS (post-op day 4) and the frequency/duration of IPC sessions (1–2 times/day, 15–20 min) could be adjusted based on patient tolerance and limb condition, as documented. The choice and dose of anticoagulant were at the treating physician’s discretion based on individual patient factors.

(B) *Core Component 2*: Perioperative nutritional support. This component was managed by nurses trained in nutritional support.

*Staff training*: Nurses completed a 2-h module on perioperative nutrition, diet calculation, and administration/monitoring of oral nutritional supplements (ONS).

*Intervention protocol*: All patients were counseled on a high-protein, high-vitamin diet.

*Intervention group specific*: Patients received a standardized oral enteral nutrition suspension (TPF, Nutricia). The regimen was fixed: Start at 100 mL, five times daily on postoperative day 1, and titrate to a fixed maintenance dose of 200 mL five times daily (1,000 mL/day) by day 7.

*Flexibility/Adaptation*: The flavor of the ONS could be changed based on patient preference to improve adherence. The feeding schedule could be slightly adjusted (e.g., dividing doses) in case of gastrointestinal intolerance.

*Management of deviations*: For nausea/vomiting, the feeding rate was slowed or ONS was diluted. For suspected aspiration, feeding was stopped immediately, and the physician was notified. Nutritional intake targets (standard weight × 30 kcal/kg/day) could be adjusted by the dietitian if significant weight loss or laboratory signs of malnutrition were observed.

#### 2.6.2 Intervention Fidelity and adherence monitoring

To ensure the intervention was delivered as intended, the following monitoring strategies were implemented:

*Nursing protocol adherence*: For the preventive nursing component, a checklist was integrated into the electronic nursing record. Nurses documented the completion of each risk-category-specific activity (e.g., education session conducted, video viewed, pump therapy applied) during each shift. Random audits of 20% of records were performed weekly by the head nurse.

*Nutritional support adherence*: For the nutritional component, a daily intake log was maintained, recording the volume of ONS consumed at each administration. Nurses documented reasons for any missed or partial doses.

*Staff performance*: Nurse competency was assessed pre-study via certification. During the study, the head nurse conducted direct observation of nursing assessments and patient education sessions for each participating nurse at least once per month to ensure protocol compliance.

*Contamination prevention*: To minimize contamination between groups, control and intervention group patients were assigned to different nursing teams whenever possible. Nursing team assignments were fixed for the study duration.

#### 2.6.3 Standard perioperative anticoagulation management (applicable to both groups)

To provide full transparency regarding the thromboprophylaxis background, we detail the standard pharmacological practices at our institution during the study period. For all patients, the decision regarding anticoagulation was made by the treating orthopedic surgeon or anesthesiologist, reflecting real-world clinical practice without a uniform enforced protocol.

*Agent selection*: The choice among LMWH (enoxaparin), fondaparinux, or warfarin was based on physician preference, patient’s renal function, bleeding risk assessment, and cost considerations.

*Dosing*: For enoxaparin, a fixed dose of 20 mg or 40 mg SC daily was common. Fondaparinux was dosed at 2.5 mg SC daily. Warfarin dosing was titrated to an INR of 2.0–3.0. Dose adjustments for renal impairment or extreme body weight were not standardized.

*Timing of initiation*: The first dose was typically administered 6–24 h after surgery, depending on the perceived intraoperative bleeding risk and the agent chosen.

*Duration*: Prophylaxis was generally continued throughout the inpatient stay. Extended prophylaxis after discharge was recommended but not uniformly prescribed or monitored, particularly for the control group.

*Adherence monitoring in the control group*: As part of routine care, nursing staff administered the prescribed medications, but systematic documentation of missed doses or reasons for non-adherence was not part of the standard protocol. This reflects a potential gap in routine care that our structured intervention aimed to address.

### 2.7 Observation indicators

1. Fasting venous blood (3 mL) was obtained from each patient in the morning and centrifuged, and hemoglobin (Hb), prealbumin (PA), and albumin (ALB) levels were measured using enzyme-linked immunosorbent assay.
2. The time taken to get out of bed and the length of hospital stay were recorded for both groups.
3. The Short Form-36 Health Survey was used to assess the patients’ quality of life (19) and included five dimensions: mental health, emotional function, social function, physical pain, and physiological function. The maximum score was 100; higher scores indicated better quality of life.
4. The Harris hip score was used to assess hip function of patients based on deformity (4 points), pain (44 points), function (47 points), and range of motion (5 points), with a total score of 100; higher scores indicated better hip function (20).
5. The incidence of DVT in both groups was recorded.
6. A self-developed nursing satisfaction questionnaire was used to assess patient nursing satisfaction. Here, “patient nursing satisfaction” specifically refers to patients’ satisfaction with the overall nursing services they received during their hospitalization for hip fracture surgery, including but not limited to the care provided by nurses, the effectiveness of nursing interventions, and the quality of the nursing environment. The questionnaire was designed based on a comprehensive review of relevant literature on nursing satisfaction assessment and in-depth interviews with 20 older adult patients with hip fractures and 10 experienced nurses in our hospital. It consists of three main dimensions: Nursing service attitude: This dimension includes 10 questions that evaluate the nurses’ communication skills, politeness, responsiveness to patients’ needs, and the level of empathy shown during care. For example, questions such as “Did the nurses communicate with you in a clear and understandable way?” and “Were the nurses polite and respectful when providing care?” are included. Each question is scored on a 5-point Likert scale, ranging from 1 (strongly disagree) to 5 (strongly agree); Nursing intervention effectiveness: There are 8 questions in this dimension that focus on the effectiveness of the nursing measures implemented during the perioperative period. These questions cover aspects like the management of pain, prevention of complications (such as deep venous thrombosis and pressure ulcers), and assistance with daily activities. For instance, “How satisfied are you with the pain management provided by the nurses?” and “Did the nurses effectively help you prevent complications?” are part of this dimension. Similarly, each question is scored from 1 to 5; Nursing environment quality: This dimension contains 7 questions that assess the cleanliness, comfort, and safety of the nursing environment. Questions like “Is the ward clean and tidy?” and “Do you feel safe in the ward?” are included, also scored on a 5-point Likert scale. The total score of the questionnaire is 100, which is calculated by summing up the scores of all the questions in the three dimensions. A score > 82 indicated “very satisfied;” a score in the range of 72–82 indicated “satisfied,” and a score < 72 indicated “not satisfied.” Satisfaction was calculated as: (very satisfied cases + satisfied cases) ÷ number of observed cases. The reliability and validity of the questionnaire were tested before its use in this study. The Cronbach’s alpha coefficient for the whole questionnaire was 0.85, indicating good internal consistency. The content validity was evaluated by a panel of 5 experts in the field of nursing, and all items were considered relevant and appropriate for assessing patient nursing satisfaction.

### 2.8 Statistical analysis

SPSS 20.0 was used for statistical analysis. Categorical data were expressed as frequency and percentage, and the *χ*^2^ test was used for group comparisons. Shapiro–Wilk test was used to detect the normality of data. Measurement data that conformed to a normal distribution were expressed as mean ± standard deviation. For inter-group comparisons of normally distributed measurement data, an unpaired *t*-test was used when comparing two independent groups. When comparing pre- and post- treatment values within the same group (paired data), a paired *t*-test was used. If measurement data did not conform to a normal distribution, non-parametric tests were used. For inter-group comparisons, the Mann–Whitney U test was applied. For paired non-parametric data, the Wilcoxon signed-rank test was used. The 95% confidence interval (CI) for the effect size was calculated. The accuracy of the Caprini risk assessment scale was evaluated using receiver operating characteristic (ROC) curve analysis. The 95% CI for the AUC was also reported to provide an estimate of the precision of the AUC value. To address the potential influence of confounding factors on the primary outcomes, multivariable logistic regression analyses were performed. The results are reported as adjusted odds ratios (AORs) with 95% CIs. Statistical significance was set at *p* < 0.05 for all tests.

## 3 Results

### 3.1 General data between the DVT group and non-DVT group

A total of 112 older adult patients were included in this study, comprising 46 males and 66 females, aged 60–85 years, with a mean age of 71.85 ± 7.53 years. No statistically significant differences in baseline characteristics existed between the two groups (*p* > 0.05; Table 1).

**Table 1:** General data between the DVT group and non-DVT group.

Items	DVT group (*n* = 38)	Non-DVT group (*n* = 74)	*t*/*χ*^2^	*p*
Gender (male/female)	16/22	30/44	0.025	0.873
Age (years)	72.42 ± 7.43	71.21 ± 7.32	0.820	0.414
BMI (kg/m^2^)	24.61 ± 2.46	24.25 ± 2.32	0.747	0.457
History of hypertension (yes/no)	20/18	36/38	0.159	0.689
History of coronary heart disease (yes/no)	14/24	25/49	0.103	0.747
History of diabetes (yes/no)	13/25	23/51	0.112	0.737
History of DVT (yes/no)	10/28	16/58	0.310	0.577

DVT, deep venous thrombosis.

### 3.2 Caprini risk assessment scale score and risk grade

Table 2 shows that the Caprini risk assessment scale score was higher in the DVT group than that in the non-DVT group (*p* < 0.05). The proportion of extremely high-risk patients in the DVT group (97.37%) was higher than that in the non-DVT group (78.37%) (*p* < 0.05).

**Table 2:** The validated original Caprini risk assessment scale score and risk grade between the two groups.

Groups	*n*	Caprini score	Caprini score classification			
			Low risk (0–1 point)	Medium risk (2 points)	High risk (3–4 points)	Highest Risk (≥5 points)
DVT group	38	8.26 ± 2.85	0	0	1 (2.63)	37 (97.37)
Non-DVT group	74	5.21 ± 1.54	0	1 (1.35)	15 (20.27)	58 (78.38)
*t*/*χ*^2^		7.364	6.521			
*p*		<0.001	0.038			

DVT, deep venous thrombosis.

### 3.3 ROC curve analysis of Caprini risk assessment scale

**Figure 2:**
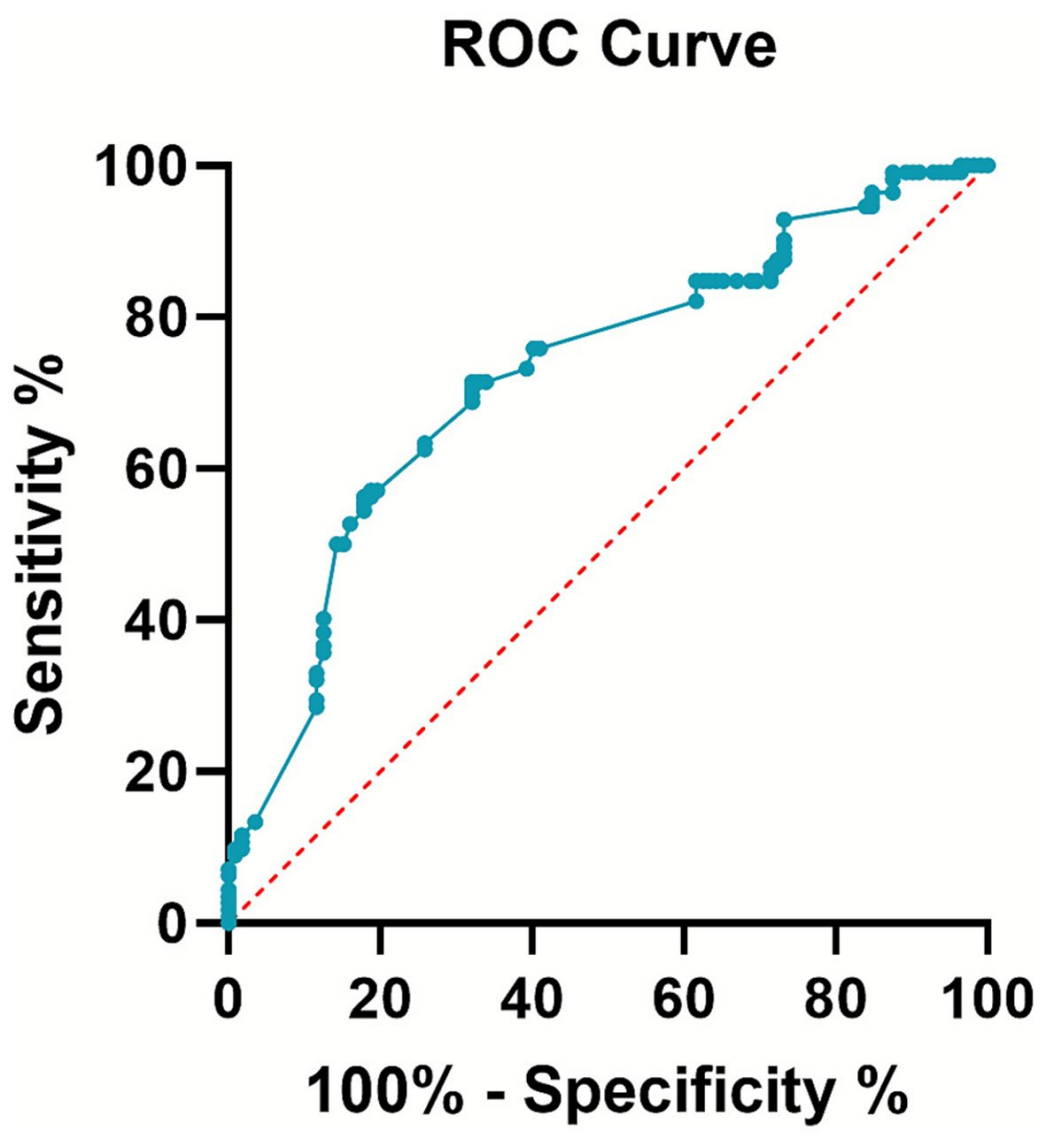
Receiver operating characteristic (ROC) curve analysis of the Caprini risk assessment scale.

The Caprini risk assessment scale score was used as the test variable, and the occurrence of DVT was the outcome variable. Figure 2 shows that the area under the curve (AUC) of the ROC curve was 0.724 with a 95% confidence interval of 0.657–0.791 (*p* < 0.001).

### 3.4 Multivariable analysis of Caprini score and DVT risk

To account for potential confounding factors, we performed a multivariable logistic regression analysis with the occurrence of postoperative DVT as the dependent variable. The model included the following covariates selected based on clinical relevance and univariate associations (*p* < 0.10): age (continuous), fracture type (femoral neck vs. intertrochanteric, as categorical), and the original Caprini risk category (using “Highest Risk: ≥5” as the reference group compared to combined “Low/Moderate/High Risk: 0–4” due to sample distribution). Anticoagulation regimen was not included as an independent variable in this model because it was part of the standard perioperative protocol and its administration was highly correlated with the Caprini risk level as per clinical guidelines.

The results showed that after adjusting for age and fracture type, patients classified in the “Highest Risk” (Caprini ≥5) category had significantly higher odds of developing DVT compared to those in the lower risk categories (Adjusted Odds Ratio [AOR] = 8.42, 95% CI: 1.78–39.87, *p* = 0.007). Age and fracture type were not independently associated with DVT risk in this adjusted model (*p* > 0.05). When entering the continuous Caprini score into a separate adjusted model, each one-point increase in the Caprini score was associated with an AOR of 1.18 (95% CI: 1.05–1.33, *p* = 0.005) for DVT, confirming the independent predictive value of the score (Table 3).

**Table 3:** Multivariable logistic regression analysis for factors associated with postoperative DVT (Phase 1).

Variable	Category/Unit	Adjusted odds ratio (AOR)	95% confidence interval	*p*-value
Caprini risk category	Highest risk (≥5) vs. Lower risk (0–4)	8.42	1.78–39.87	0.007
Age	Per year increase	1.03	0.96–1.11	0.385
Fracture type	Intertrochanteric vs. femoral neck	1.92	0.79–1.33	0.151
Caprini score	Per point increase	1.18	1.05–1.33	0.005

### 3.5 General data between the control group and intervention group

No statistically significant differences in baseline characteristics existed between the intervention group and control group (*p* > 0.05; Table 4).

**Table 4:** General data between the control group and intervention group.

Items	Control group (*n* = 56)	Intervention group (*n* = 56)	*t*/*χ*^2^	*p*
Gender (male/female)	22/34	24/32	0.14	0.70
Age (years)	72.36 ± 7.35	72.58 ± 7.46	0.15	0.87
BMI (kg/m^2^)	24.53 ± 2.52	24.47 ± 2.43	0.12	0.89
History of hypertension (yes/no)	30/26	26/30	0.57	0.44
History of coronary heart disease (yes/no)	19/37	20/36	0.03	0.84
History of diabetes (yes/no)	20/36	16/40	0.65	0.41
History of DVT (yes/no)	14/42	12/44	0.20	0.65

DVT, deep venous thrombosis.

### 3.6 Nutritional status

**Figure 3:**
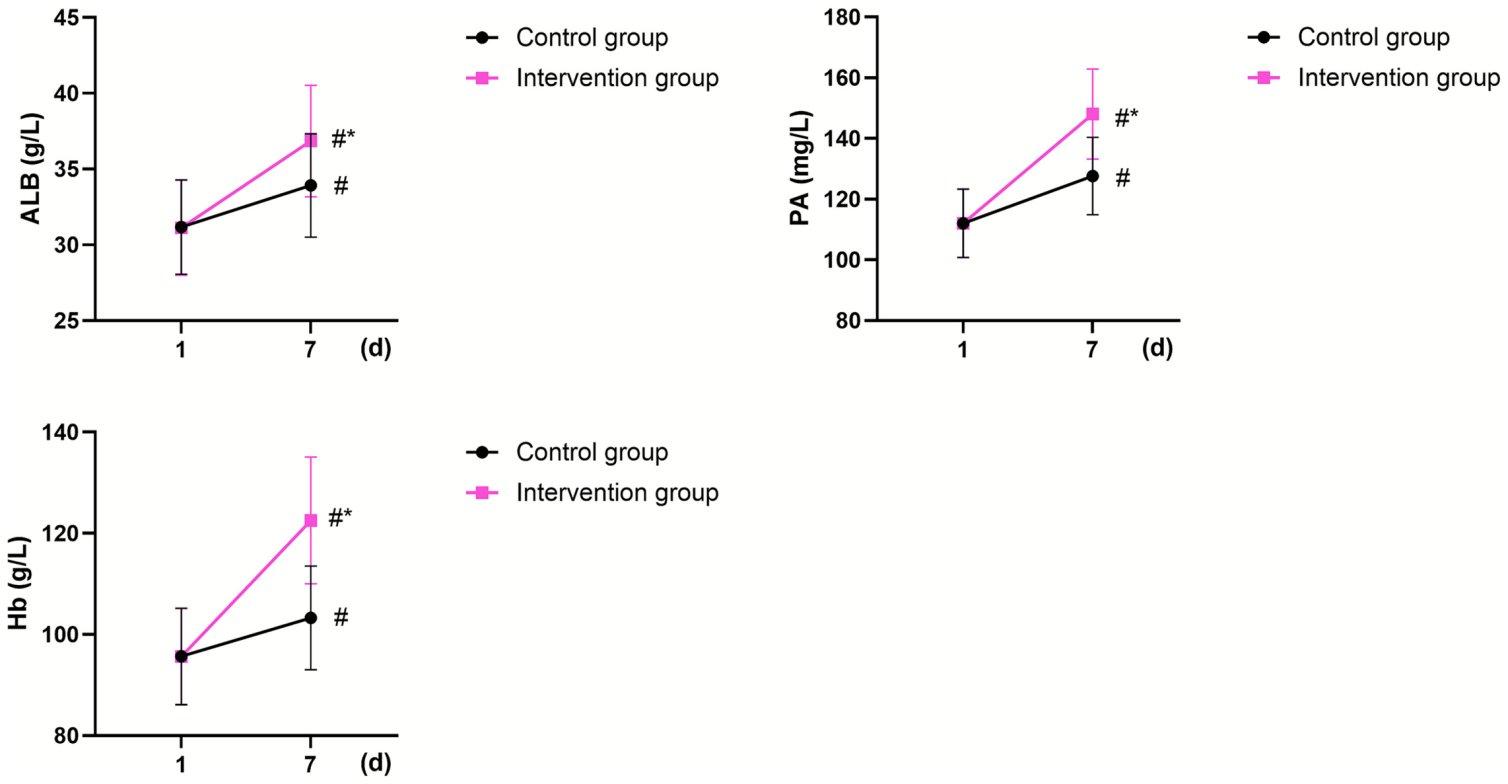
Nutritional status in the two groups. ^#^Indicates a statistically significant difference compared with preoperative values, with *p* < 0.05. *Indicates a statistically significant difference compared with the CG, with *p* < 0.05.

On postoperative day 1, no significant differences were observed in ALB, PA, and Hb levels between the two groups (*p* > 0.05). On postoperative day 7, ALB, PA, and Hb increased in both groups (*p* < 0.05, 95% CI: −2.320–0.559; *p* < 0.05, 95% CI: −13.530–6.892; *p* < 0.05, 95% CI: −12.39–6.847;), with significantly higher levels in the intervention group than those in the control group (*p* < 0.05, 95% CI: −5.120–3.360; *p* < 0.05, 95% CI: −29.10–22.46; *p* < 0.05, 95% CI: −20.04–14.50; Figure 3).

### 3.7 Time of getting out of bed and hospital stay

**Figure 4:**
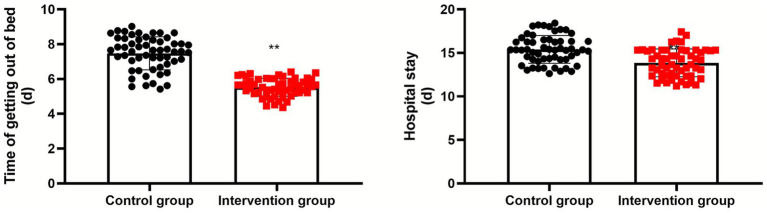
Time to immobilization and length of hospital stay in the two groups. **Indicates a highly statistically significant difference between the two groups, with *p* < 0.01.

Compared with the control group, the intervention group had a shorter time of getting out of bed and hospital stay (*p* < 0.01, 95% CI: −2.234–1.662; *p* < 0.01, 95% CI: −2.145–0.948; Figure 4).

### 3.8 Quality of life

**Figure 5:**
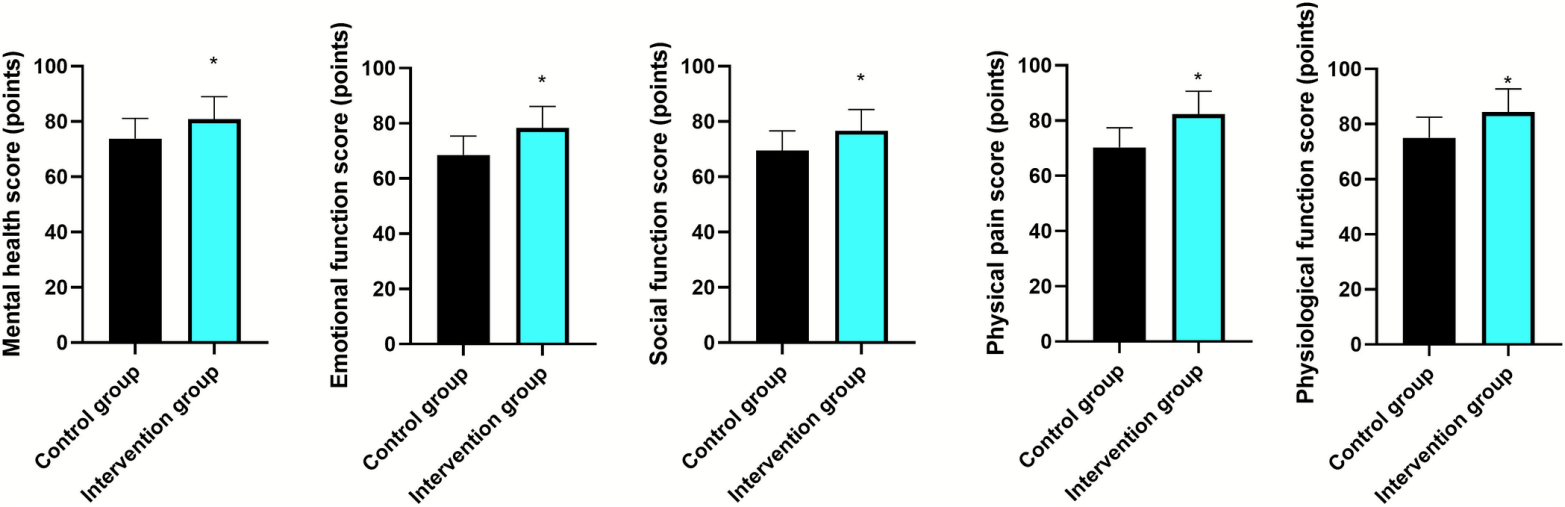
Quality of life in the two groups. *Indicates a highly statistically significant difference between the two groups, with *p* < 0.05.

After nursing, the scores of mental health, emotional function, social function, physical pain, and physiological function were higher in the intervention group than those in the control group (*p* < 0.05; 95% CI: 4.246–10.03; *p* < 0.05; 95% CI: 7.005–12.52; *p* < 0.05; 95% CI: 4.365–9.875; *p* < 0.05; 95% CI: 9.168–14.91; *p* < 0.05; 95% CI: 6.312–12.29; Figure 5).

### 3.9 Hip function

**Figure 6:**
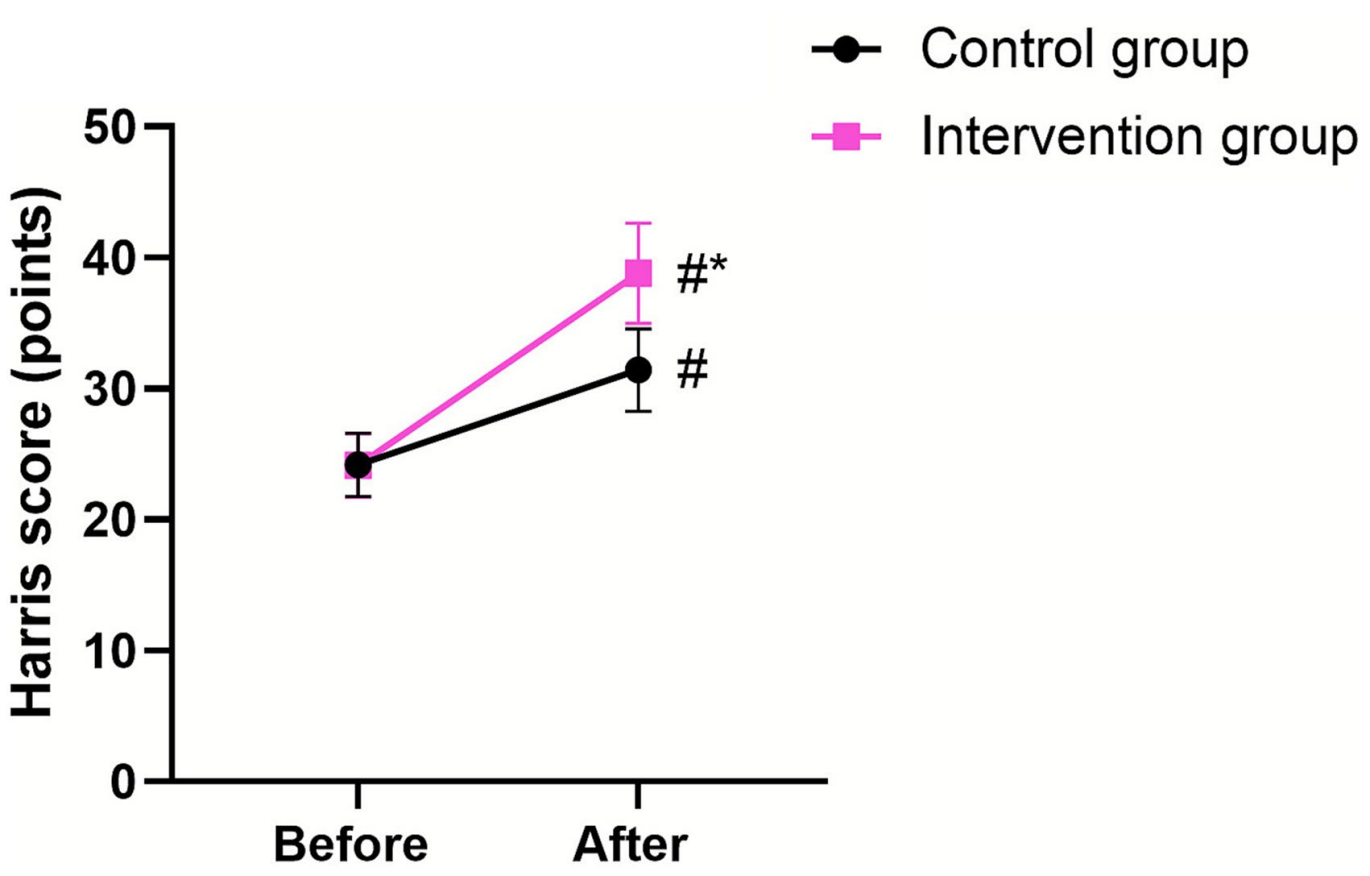
Hip function in the two groups. ^#^Indicates a statistically significant difference compared with preoperative values, with *p* < 0.05. *Indicates a statistically significant difference compared with the CG, with *p* < 0.05.

Before surgery, no significant difference was observed in the Harris hip score between the two groups (*p* > 0.05). At discharge, the score increased in both groups (*p* < 0.05, 95% CI: −4.480–2.890), with the intervention group scoring higher than the control group (*p* < 0.05, 95% CI: −11.75–10.16; Figure 6).

### 3.10 Incidence of DVT and adjusted analysis

Table 5 shows that the incidence of DVT was 17.8% (10/56) in the intervention group compared to 50% (28/56) in the control group (*p* < 0.05).

**Table 5:** Incidence of deep venous thrombosis (DVT) in the two groups.

Groups	*N*	DVT (cases)	DVT (%)
Control group	56	28	50.00
Intervention group	56	10	17.85
*χ* ^2^		12.90	
*p*		< 0.001	

DVT, deep venous thrombosis.

To assess the robustness of this association while considering potential baseline imbalances, a multivariable logistic regression was performed for this RCT phase outcome. The dependent variable was DVT occurrence. The primary independent variable was group assignment (Intervention vs. Control). Covariates included age, fracture type, and the baseline Caprini score (continuous) to adjust for any residual differences in baseline risk despite randomization.

The adjusted analysis confirmed that assignment to the intervention group remained a strong, independent predictor of lower DVT odds after controlling for these factors (AOR = 0.16, 95% CI: 0.06–0.42, *p* < 0.001). The baseline Caprini score was also independently associated with DVT risk (AOR per point = 1.22, 95% CI: 1.08–1.37, *p* = 0.001), while age and fracture type were not significant in this model (Table 6).

**Table 6:** Adjusted analysis of intervention effect on DVT incidence (Phase 2, RCT).

Variable	Category/Unit	Adjusted odds ratio (AOR)	95% confidence interval	*p*-value
Group assignment	Intervention vs. Control	0.16	0.06–0.42	<0.001
Baseline Caprini score	Per point increase	1.22	1.08–1.37	0.001
Age	Per year increase	1.02	0.95–1.10	0.567
Fracture type	Intertrochanteric vs. femoral neck	1.65	0.63–4.35	0.309

### 3.11 Nursing satisfaction

Table 7 shows that nursing satisfaction was higher in the intervention group than that in the control group (*p* < 0.05).

**Table 7:** Nursing satisfaction in the two groups.

Groups	*n*	Very satisfied	Satisfied	Dissatisfied	Total satisfaction rate
Control group	56	26	20	10	46 (82.14%)
Intervention group	56	30	24	2	54 (96.42%)
*χ* ^2^					5.973
*p*					0.014

## 4 Discussion

Older adults often have poor bone quality, low bone mass, high bone fragility, diminished muscles and ligament responsiveness, and a weak ability to prevent falls. Consequently, even minor trauma can lead to immediately fractures (21). In addition, they frequently have multiple comorbidities, reduced physical function, poor tolerance to surgery after hip fracture, and a higher risk of various postoperative complications, which may have a great impact on patient outcomes than the fracture itself (22). DVT is one of the most common complications of orthopedic surgery, and if not treated promptly, it may cause pulmonary embolism, cerebral infarction, and other life-threatening conditions (23). Therefore, patients undergoing fracture surgery—especially older adult patients—should be closely monitored for DVT after hip fracture surgery.

The Caprini risk assessment scale is a widely used clinical tool for assessing DVT risk. It evaluates factors that may cause DVT and assigns scores to estimate the likelihood of occurrence, demonstrating high reliability (24). By using the Caprini risk assessment table to stratify patients according to risk level, targeted preventive nursing interventions can be implemented, avoiding the nonspecific nature of routine care and improving both nursing efficiency and quality (25).

Our results showed that the original Caprini risk assessment scale score in the DVT group was higher than that in the non-DVT group. More importantly, when applying the original, validated risk classification, a significantly higher proportion of patients in the DVT group (97.37%) fell into the “highest risk” category (score ≥5) compared to the non-DVT group (78.38%). This finding reinforces the utility of the original model in distinguishing risk within this specific surgical population. The ROC curve analysis showed an AUC of 0.724, indicating moderate predictive accuracy. This analysis, based on the continuous score from the original model, confirms its predictive validity without reliance on unvalidated modifications. Furthermore, a multivariable logistic regression analysis, adjusting for age and fracture type, confirmed that being classified in the ‘Highest Risk’ category (Caprini ≥5) was independently associated with an over eight-fold increase in the odds of developing DVT. This underscores that the predictive capacity of the Caprini model, as used in its original form, holds significant clinical relevance even after accounting for other patient characteristics.

Regarding the intervention framework, the preventive nursing measures were applied based on the risk strata defined by this original Caprini model. The significant reduction in DVT incidence observed in the intervention group can therefore be attributed to a nursing strategy guided by this standardized, validated risk assessment tool.

Malnutrition is not only a risk factor for hip fracture but also a predictor of mortality within the first year after fracture (26). In older adult patients with hip fractures, trauma, stress, and other factors increase the body’s energy consumption, and protein breakdown. Chronic inflammation after surgery, insulin resistance, and other conditions further reduce the body’s sensitivity to essential amino acids, leading to decreased protein synthesis (27). Poor nutrition status can prolong hospital stay; increase the risk of complications such as nosocomial infection, sepsis, and pressure ulcers; and raise the incidence of postoperative delirium (28). Therefore, improving nutritional status is essential for optimizing outcomes in older adult patients with hip fractures.

In our study, on postoperative day 7, ALB, PA, and Hb levels in the intervention group group were higher than those in the control group, suggesting that perioperative nutritional support combined with preventive nursing (guided by the Caprini risk assessment) improved the nutritional status of older adult patients undergoing hip fracture surgery. Targeted nutritional intervention combined with early enteral nutrition can significantly reduce the burden of gastrointestinal peristalsis and meet patient’s daily nutritional needs, thereby improving nutritional outcomes (29).

Our study observed a notable reduction in DVT incidence, with 17.8% in the intervention group compared to 50% in the control group. The significant reduction in DVT incidence observed in the intervention group (17.8% vs. 50%) persisted in a more rigorous, multivariable analysis. After adjusting for baseline age, fracture type, and the pre-randomization Caprini score—a key indicator of intrinsic DVT risk—assignment to the combined intervention remained a strong, independent protective factor. This adjusted analysis strengthens the inference that the observed benefit is likely attributable to the intervention itself, rather than to uneven distribution of baseline risk profiles between the randomized groups.

In addition, compared with the control group, the intervention group had shorter time to time to immobilization and hospital stay, higher scores for mental health, emotional function, social function, physical pain, and physiological function, higher Harris hip scores, and higher nursing satisfaction. These findings suggest that perioperative nutritional support combined with Caprini-based preventive nursing can promote postoperative recovery and hip function, improve quality of life, and enhance nursing satisfaction in older adult patients undergoing hip fracture surgery—consistent with previous research (30–33).

However, when interpreting these positive results, especially the significant reduction in the incidence of DVT (50% vs. 17.8%), we must carefully consider other possible explanations. Firstly, monitoring bias may play a role: the intervention group may have a higher detection rate of DVT due to receiving a more intensive care plan (including regular assessments based on the Caprini score). However, in this study, both groups received a unified ultrasound screening protocol, aiming to minimize such bias. Secondly, the difference in the intensity of care is an inherent part of the intervention design, intended to evaluate the effect of structured and individualized care plans. The multi-modal preventive measures (such as physical prevention and enhanced assessment) received by the intervention group are precisely what the study is attempting to test. Thirdly, the issue of the blinding of intervention implementation does indeed have limitations: both the nurses and the patients were aware of the group allocation, which may affect the results through the Hawthorne effect or differences in the behavior of the nurses. We acknowledge this as a potential source of bias. Fourthly, the imbalance in anticoagulant treatment is an important factor to consider. Although all patients received drug prophylaxis according to hospital standards, there may be subtle differences in the timing of administration, dosage, or compliance. Future studies should detail and compare the anticoagulant treatment regimens of the two groups to control this potential confounding factor.

However, our research also has several limitations that need to be acknowledged.

First, the sample size in our study was relatively small. This limited sample may not fully represent the entire population of older adult patients with hip fractures, thereby affecting the generalizability of our findings. Future studies should aim to recruit a larger number of patients from multiple centers across different geographical regions. This multi-center approach would not only increase the sample size but also incorporate a more diverse patient population with varying clinical characteristics and healthcare practices, thus enhancing the external validity of the research.

Second, regarding the multivariable logistic regression analysis performed in Phase 1, we acknowledge its statistical limitations. With 38 observed DVT events and the inclusion of multiple covariates (age, fracture type, and the ‘Highest Risk’ category), the events-per-variable (EPV) ratio is relatively low. This can lead to model instability, overfitting, and potentially inflated effect estimates, such as the reported eight-fold increase in DVT odds. While the association remains statistically significant and clinically plausible, the precision of the effect size should be interpreted with caution. Future studies with larger cohorts are needed to validate this association with greater statistical robustness. Meanwhile, we adjusted for the baseline Caprini score in our Phase 2 model to account for residual risk imbalance. While this is methodologically sound for a baseline covariate, we acknowledge that the score also informed the intervention intensity for the intervention group. Our primary interpretation focuses on the overall effect of the bundled intervention strategy, which includes risk-stratified care.

Third, our study was conducted in a single hospital. This single-center design is prone to selection bias, as the patient population in this specific hospital may have unique characteristics that are not representative of the broader population of older adults with hip fractures. For example, the hospital’s referral patterns, admission criteria, and local demographic factors could influence the types of patients included in the study. Consequently, our findings may not be applicable to other healthcare settings with different patient populations and clinical practices. Future multi-center studies involving patients from diverse geographical areas and medical centers are essential to minimize selection bias and improve the generalizability of the research.

Fourth, we did not conduct long-term follow-up to assess the sustained impact of perioperative nutritional support combined with preventive nursing on patient outcomes. Our study primarily focused on short-term outcomes, such as nutritional status and DVT incidence during the immediate postoperative period. However, the long-term benefits and potential risks of these intervention measures, such as the impact on functional recovery, quality of life, and the recurrence of DVT or other complications over an extended period, remain unknown. Longer-term follow-up studies are needed to comprehensively evaluate the effectiveness and safety of these interventions in older adult patients undergoing hip fracture surgery.

Fifth, we utilized a self-developed nursing satisfaction questionnaire to evaluate patient nursing satisfaction among hip fracture surgery patients. While we reported the Cronbach’s alpha coefficient (0.85) to demonstrate the internal consistency reliability of the questionnaire, we acknowledge that there was no construct validity assessment, no comparison with standard instruments, unclear scoring rationale and lack of factor analysis. These limitations may affect the objectivity and consistency of satisfaction classification, and highlight the need for further validation of this self-developed questionnaire before it can be confidently used as a major outcome measure in future research.

Finally, we acknowledge that a formal assessment of the optimal score threshold within the “highest risk” category (≥5) for this specific population was not performed. Future studies could employ ROC-derived cut-offs within this high-risk stratum to further refine risk prediction.

In conclusion, our study demonstrates that the original Caprini risk assessment model has predictive value for DVT risk in older adult patients with hip fractures. Furthermore, the combined strategy of perioperative nutritional support and preventive nursing, guided by this model, was associated with improved nutritional status and a significantly reduced incidence of DVT in this population. Positive trends in functional recovery and length of hospital stay were also observed. Given the lack of external validation for the self-developed nursing satisfaction questionnaire, we refrain from drawing strong conclusions based on this measure alone. Future research employing multicenter designs, longer follow-up, and validated outcome instruments is warranted to confirm the efficacy and generalizability of this combined intervention strategy before broad clinical implementation can be recommended.

## Data Availability

The datasets presented in this study can be found in online repositories. The names of the repository/repositories and accession number(s) can be found in the article/supplementary material.
